# CCR9 Expressing T Helper and T Follicular Helper Cells Exhibit Site-Specific Identities During Inflammatory Disease

**DOI:** 10.3389/fimmu.2018.02899

**Published:** 2019-01-04

**Authors:** Ilaria Cosorich, Helen M. McGuire, Joanna Warren, Mark Danta, Cecile King

**Affiliations:** ^1^Department of Immunology, The Garvan Institute of Medical Research, Darlinghurst, NSW, Australia; ^2^St Vincent's Clinical School, University of NSW, Sydney, NSW, Australia

**Keywords:** CCR9 C-C chemokine receptor type 9, T follicular helper, T helper, inflammation, GIT = gastrointestinal tract, autoimmunity

## Abstract

CD4^+^ T helper (Th) cells that express the gut homing chemokine receptor CCR9 are increased in the peripheral blood of patients with inflammatory bowel disease and Sjögren's syndrome and in the inflamed lesions of autoimmune diseases that affect the accessory organs of the digestive system. However, despite the important role of the GIT in both immunity and autoimmunity, the nature of CCR9-expressing cells in GIT lymphoid organs and their role in chronic inflammatory diseases remains unknown. In this study, we analyzed the characteristics of CCR9^+^ Th and T follicular helper (Tfh) cells in GIT associated lymphoid tissues in health, chronic inflammation and autoimmunity. Our findings reveal an association between the transcriptome and phenotype of CCR9^+^ Th in the pancreas and CCR9^+^ Tfh cells from GIT-associated lymphoid tissues. GIT CCR9^+^ Tfh cells exhibited characteristics, including a Th17-like transcriptome and production of effector cytokines, which indicated a microenvironment-specific signature. Both CCR9^+^ Tfh cells and CCR9^+^ Th cells from GIT-associated lymphoid tissues migrated to the pancreas. The expression of CCR9 was important for migration of both subsets to the pancreas, but Tfh cells that accumulated in the pancreas had downmodulated expression of CXCR5. Taken together, the findings provide evidence that CCR9^+^ Tfh cells and Th cells from the GIT exhibit plasticity and can accumulate in distal accessory organs of the digestive system where they may participate in autoimmunity.

## Introduction

### T Cell Mediated Damage to Self-Tissue

It was demonstrated over four decades ago that experimental, partial depletion of T cells precipitates self-tissue destructive inflammation (1, 2). Subsequent to these studies, a T cell transfer model of colitis was described in rats and then in mice. These studies showed that transfer of antigen naïve (CD45RB^hi^) CD4^+^ T cells into immunodeficient recipients led to colitis (3). The transferred T cells proliferated within the lymphopenic host, inducing effector functions that could be prevented by cotransfer of memory phenotype (CD4^+^CD45RB^low^) T cells (3) and, more specifically, FoxP3^+^ CD4^+^ T regulatory (Treg) cells (4). Importantly, transfer of naïve CD4^+^ T cells into germ free mice does not induce colitis indicating that inflammation in the gut results from a dysregulated immune response toward commensal microbes (5).

The gastrointestinal tract (GIT) contains immunological inductive sites, such as the Peyer's patches (PP), organized lymphoid aggregates and the adjacent mesenteric lymph nodes (MLN). The immune cells at these sites coordinate the balance between tolerance to food antigens and commensal microbes, whilst ensuring effective immune responses to mucosal pathogens. In humans, the chronic inflammatory bowel diseases (IBD), which include Crohn's disease (CD) and ulcerative colitis (UC), affect approximately 0.3% of the Western population (6) with increasing incidence worldwide (7). Antibody-neutralization studies have implicated cytokines (including tumor necrosis factor alpha (TNFα) and IL-12 p40) in the pathogenesis of CD, while the effectiveness of T-cell-ablative therapies have implicated T cells in UC (8). Bacteria reactive T lymphocytes have been more frequently observed in patients with IBD than in healthy individuals (9), leading to the proposal that a breakdown of tolerance toward the intestinal microflora plays a role in the pathogenesis of IBD.

### The Gastrointestinal Tract and Chronic Inflammation

One pertinent question to the study of autoimmune and chronic inflammatory diseases is how inflammation in the GIT can influence the development of inflammation in distal tissues. Studies on a diverse array of inflammatory diseases indicate that the role of microbiota and the GIT barrier may extend well-beyond the gut (10). Accessory organs of the digestive system, connected to the small intestine by excretory ducts, include the pancreas and salivary glands, which are targets of the autoimmune diseases Type-1 diabetes (T1D) and Sjögren's Syndrome, respectively. The autoimmune disease that develops in non-obese diabetic (NOD) mice targets the pancreas and other accessory organs of the digestive system, namely, the salivary glands (11) and gallbladder (12). Antigen specificity is considered a prerequisite for the accumulation of T cells in the islet lesion (13), but the site of priming of diabetogenic T cells remains unknown. Lymphocytes infiltrating the pancreatic islets in both human T1D and NOD mice express α4β7-integrin, supporting a link between T1D and the gastrointestinal immune system (14). Furthermore, antibodies blocking either α4β7-integrin or its ligand, the mucosal addressin cell adhesion molecule (MadCAM-1), prevent diabetes in NOD mice (15, 16).

### T Helper Cells in the GIT and Associated Lymphoid Tissues

In the GIT, T-dependent antibody responses are strongly biased toward IgA, which has a crucial role modulating immune responses to commensal microbiota and neutralizing intestinal pathogens (17, 18). IL-17 producing CD4^+^ (Th17) cells that express the transcription factor RORγt and the IL-23 receptor are enriched in normal intestines. Th17 cells contribute to intestinal homeostasis by regulating IgA secretion and play a critical biological function in clearing extracellular pathogens through the release of effector cytokines (interleukin (IL); IL-17A, IL-17F, IL-21, and IL-22) (19). In this manner, Th17 cells also contribute to the pathology of inflammatory diseases, including IBD (20, 21). The production of affinity-matured antibody requires the interaction of B cells with a specialized subset of Th cells named T follicular helper (Tfh) cells (22), but the identity of Tfh cells that provide help to B cells in the GIT is only beginning to be understood.

### CCR9^+^ T Helper Cells

Retinoic acid can induce the expression of integrin α4β7 and the G protein coupled chemokine receptor 9 (CCR9) on lymphocytes, which allows their migration toward the GIT that expresses the chemokine ligand CCL25 (23). In healthy humans and mice, CCR9 is expressed predominately by a subset of T cells that migrate selectively to the gut (24, 25). Increased numbers of CCR9 expressing T cells have been observed in peripheral blood of patients with IBD (26). More recently, we described a T helper (Th) cell subset based upon expression of CCR9 (termed Tccr9 cells) that contribute to the regional specification of organ-specific autoimmune disease (27). Tccr9 cells constituted only a small fraction of CD4^+^ T cells in the lymphoid tissues and circulation of healthy mice and humans, but exhibited an inappropriate accumulation in the autoimmune lesions of the pancreas and salivary glands of NOD mice and were abundant in the peripheral blood of most Sjögren's syndrome patients. Tccr9 cells exhibited characteristics of T follicular helper (Tfh) cells-including expression of Bcl6, IL-21, c-Maf, and ICOS (27), suggesting that Tccr9 cells may be selectively recruited from a CCR9^+^ precursor population in the follicular environment of gut-associated lymphoid tissue.

In healthy humans, CCR9 is found primarily on T cells that selectively migrate to the GIT and is thought to play a role in several inflammatory disorders of the GIT. However, our studies demonstrate that during autoimmunity and chronic inflammation, CCR9^+^ T helper cells also infiltrate the pancreas and other accessory organs of the digestive system and are crucial to the destruction of these tissues. The implication of these findings is that T cells that are activated in the gut can disseminate to other organs to cause tissue damage. Here, we analyse CCR9^+^ Th and Tfh cells within the GIT and GIT associated lymphoid tissues to determine whether CCR9 expression and the characteristics of these populations reflect the state of inflammation.

## Results

### GIT Inflammation in Il2*^−/−^* Mice

Our previous studies demonstrated that the GIT-homing chemokine receptor CCR9 marked a subset of IL-21-producing Th cells in the inflamed lesions of the pancreas and salivary glands of T1D prone NOD mice (27). Examination of the phenotype of this population suggested a close relationship between CCR9^+^ Th cells and Tfh cells and we hypothesized that CCR9^+^ Th cells may emerge from Tfh-like cells in GIT lymphoid tissue. However, we had yet to analyse the characteristics of CCR9^+^ cells in the GIT and whether CCR9+ Th cells were distinct under conditions of GIT inflammation. Therefore, we examined CCR9^+/−^ Th and CCR9^+/−^ Tfh cells in two models of autoimmunity and inflammation, namely the NOD mouse and mice that have been made genetically deficient in IL-2 (*Il2*^−/−^mice). NOD mice exhibit a mild subclinical colitis (28), whereas *Il2*^−/−^ mice that lack IL-2 dependent Tregs and exhibit multi-organ autoimmunity, exhibit a chronically inflamed GIT (29). The GIT inflammation in *Il2*^−/−^ mice is influenced by microbiotia as colitis is significantly reduced under germ-free conditions (30).

**Figure 1:**
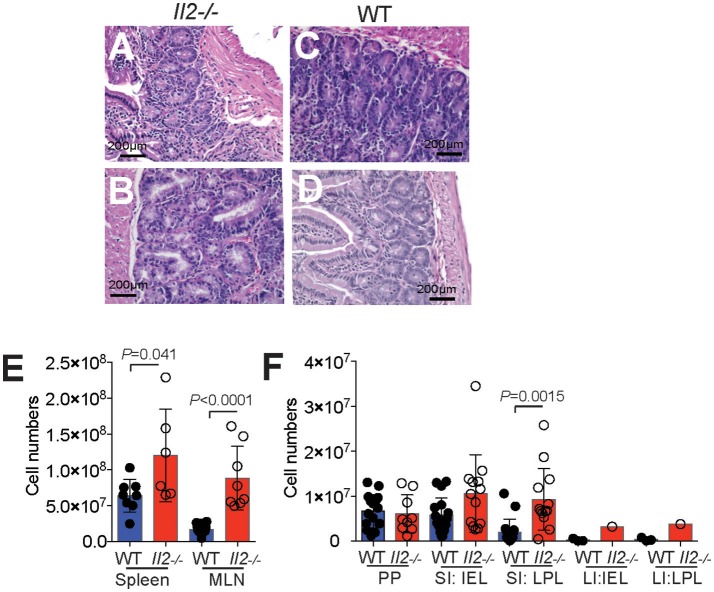
Inflamed bowel and numeration of leukocytes in *Il2*^−/−^ mice. Representative histological sections of intestinal tissues from *Il2*^−/−^ mice **(A,B)** and WT mice **(C,D)** mice stained with hematoxylin and eosin, optical microscope 40X. **(E,F)** FACS analyses and cell counts were used to determine numbers of CD45^+^ cells in *Il2*^−/−^ and WT mice, cell numbers were normalized to organ weight. Data shown as mean ± SEM (*n* = 3-9) and analyzed by Students *T*-test.

Histological analyses of the intestine of *Il2*^−/−^ and WT mice stained with hematoxylin and eosin showed increased leukocyte infiltration of the intestine of *Il2*^−/−^ mice (Figures 1A,B) compared with WT mice (Figures 1C,D). There were greater numbers of leukocytes (index of inflammation) in the GIT of *Il2*^−/−^ mice; increased numbers of CD45^+^ leukocytes in the spleen, mesenteric lymph nodes (MLN) (Figure 1E) and increased numbers of lamina propria lymphocytes (LPL) in the small intestine (SI LPL) compared with WT mice as shown by FACS analysis (Figure 1F). In the large intestine, there was a trend of increased numbers of LPL and intraepithelial lymphocytes (IEL) in *Il2*^−/−^ mice relative to WT mice (Figure 1F). By contrast, there were no differences observed in the numbers of CD45^+^ leukocytes in the Peyer's Patches (PP) or small intestine intraepithelial lymphocytes (SI IEL) (Figure 1F).

### Increased Numbers of CCR9^+^ Th and Tfh Cells in the Inflamed GIT

**Figure 2:**
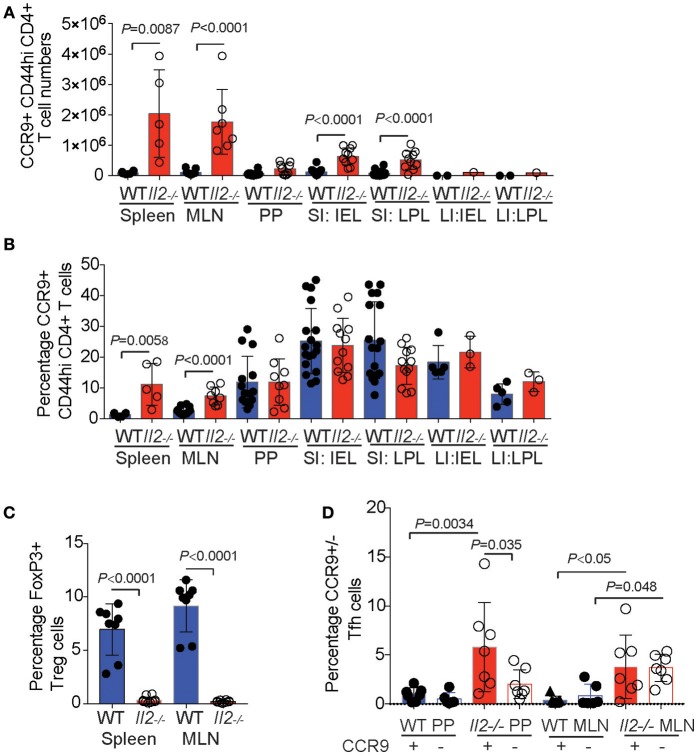
Expansion of CCR9^+^ CD4^+^ T cells in *Il2*^−/−^ mice. Numeration **(A)** and percentages **(B)** of CCR9^+^ and CCR9^−^ CD44^hi^ CD4^+^ T cells in *Il2*^−/−^ and WT mice determined by FACS analysis. (MLN) mesenteric lymph nodes; (PP) Peyer's Patches; (SI IEL) intraepithelial lymphocytes of the small intestine; (SI LPL) lamina propria lymphocytes of the small intestine; (LI IEL) intraepithelial lymphocytes of the large intestine; (LI LPL) lamina propria lymphocytes. **(C)** Percentages of FoxP3^+^ CD4^+^ TCRb^+^ T regulatory (Treg) cells in the spleen and MLN and **(D)** percentages of CXCR5^+^ PD1^+^ FoxP3^−^ CD4^+^ TCRb^+^ T follicular helper (Tfh) cells in the PP and MLN of *Il2*^−/−^ and WT mice. Data shown as mean ± SD, where *n* = 3-15 female mice 7-9 weeks of age. Statistical significance was analyzed by students *T*-test.

We next examined the numbers of CD44^hi^ (activated/memory phenotype) CD4^+^ T cells expressing CCR9 compared with those that lack CCR9 expression across GIT and GIT-associated lymphoid tissues. The results demonstrate greater numbers of CCR9^+^ CD44^hi^ T cells in the inflamed GIT of *Il2*^−/−^ mice compared with WT mice in the spleen, MLN, SI IEL, SI LPL, but not in PP or LI IEL or LI LPL where the low cell numbers retrieved at these sites influenced group numbers (Figure 2A). When analyzed as a percentage of CD4^+^ CD44^hi^ T cells, CCR9^+^ cells were increased in the spleen and MLN of *Il2*^−/−^ mice relative to WT mice (Figure 2B). However, there were no statistical differences in the percentages of CCR9^+^ cells within the CD4^+^CD44^hi^ populations in the PP, SI IEL, LI IEL, LI LPL (Figure 2B). Taken together, these results show that *Il2*^−/−^ mice exhibit an expansion of CCR9^+^ Th cells, and there is a specific increase in the proportion of gut homing CCR9^+^ memory phenotype CD4^+^ T cells in the spleen, MLN and SI LPL.

Foxp3^+^ T follicular regulatory T (Tfr) cells are a specialized subset of Tregs cells that colocalize within B cell follicles of secondary lymphoid tissues and exhibit characteristics attributed to both Treg and Tfh cells, and are included within the C-X-C chemokine receptor 5 (CXCR5)^+^, programmed cell death protein (PD1)^+^ population of CD4^+^ T cells (29, 31, 32). To determine the percentages of Tfh cells in *Il2*^−/−^ and WT mice, we gated out Foxp3^+^ cells as *Il2*^−/−^ mice have a deficiency of IL-2 dependent FoxP3+ Treg cells (Figure 2C). The percentages of PD1^+^ CXCR5^+^ Foxp3^−^ Tfh cells were significantly increased in both the MLN and PP of *Il2*^−/−^ mice compared with WT mice (Figure 2D), as we have observed previously for ICOS^+^ CXCR5^+^ CD4^+^ T cells in *Il2*^−/−^ mice (29). Within the FoxP3^−^ Tfh population, CCR9^+^ Tfh cells were increased in the PP of *Il2*^−/−^ mice compared with Tfh cells that lacked CCR9 (Figure 2D).

### CCR9+ Th Cells in the GIT Exhibit Both Th17 and Th1 Characteristics

**Figure 3:**
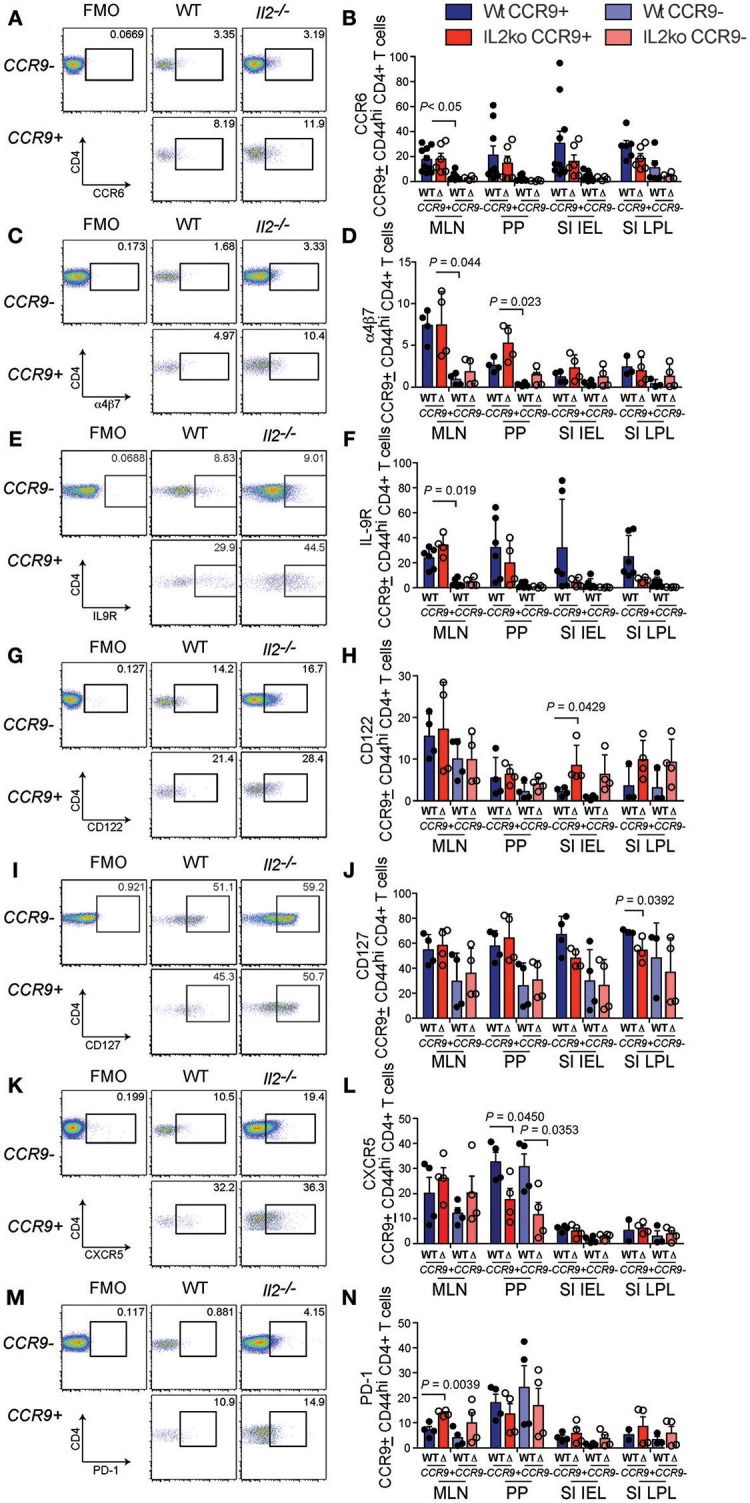
Distinct phenotypic profile of CCR9+ Th cells in the inflamed GIT. Flow cytometric analyses showing representative FACS dot plots of surface marker expression for CCR9^+^ and CCR9^−^ CD44^hi^ CD4^+^ T cells from the mesenteric lymph nodes and quantitation of the percentages of CCR9^+^ and CCR9^−^ CD44^hi^ CCD4^+^ T cells from *Il2*^−/−^ and WT mice. CCR6; **(A)** representative FACS dot plot and **(B)** quantitation. a4b7; **(C)** representative FACS dot plot and **(D)** quantitation. IL9R; **(E)** representative FACS dot plot and **(F)** quantitation. CD122; **(G)** representative FACS dot plot and **(H)** quantitation. CD127; **(I)** representative FACS dot plot and **(J)** quantitation. CXCR5; **(K)** representative FACS dot plot and **(L)** quantitation. PD1; **(M)** representative FACS dot plot and **(N)** quantitation. (MLN) mesenteric lymph nodes; (PP) Peyer's Patches; (SI IEL) intraepithelial lymphocytes of the small intestine; (SI LPL) lamina propria lymphocytes of the small intestine; (LI IEL) intraepithelial lymphocytes of the large intestine; (LI LPL) lamina propria lymphocytes of the large intestine. Data shown as mean + SD, where n = 3-9 female mice at 7-9 weeks of age. Statistical significance was assessed by students T-test.

For the initial characterization of CCR9^+^ Th cells, we analyzed the expression of the surface markers C-C chemokine receptor type 6 (CCR6), the integrin α4β7, IL-9R, the IL-2 receptor beta chain (CD122), the IL-7 receptor (CD127), CXCR5, and PD-1 on the CCR9^+^ Th population by FACs (Figure 3). There was a trend of increased expression of the chemokine receptor CCR6 on CCR9^+^ Th cells in all lymphoid organs examined in both *Il2*^−/−^ and WT mice, which reached significance in the MLN (Figures 3A,B). Similarly, α4β7 (Figures 3C,D) and IL-9R (Figures 3E,F) were consistently coexpressed with CCR9 on CD4^+^ T cells in the MLN and PP in both *Il2-*^/−^ and WT mice. The percentage of CD4^+^ T cells expressing IL-2R beta (CD122), was significantly increased on CCR9^+^ CD4^+^ T cells within the SI IEL of the *Il2*^−/−^ mice (Figures 3G,H). In contrast to *Il2*^−/−^ mice, WT mice harbored an increased percentage of CCR9^+^ CD4^+^ T cells expressing IL-7R (CD127) in the SI LPL (Figures 3I,J). Whilst the overall percentages of CCR9^+^ Tfh cells were increased in *Il2*^−/−^ mice compared with WT mice (Figure 2D), the percentages of CCR9^+^ Th cells co-expressing CXCR5 were increased in the PP of WT mice (Figures 3K,L). By contrast, the percentages of CCR9^+^ Th cells co-expressing PD-1 were increased in the MLN of *Il2*^−/−^ mice (Figures 3M,N).

**Figure 4:**
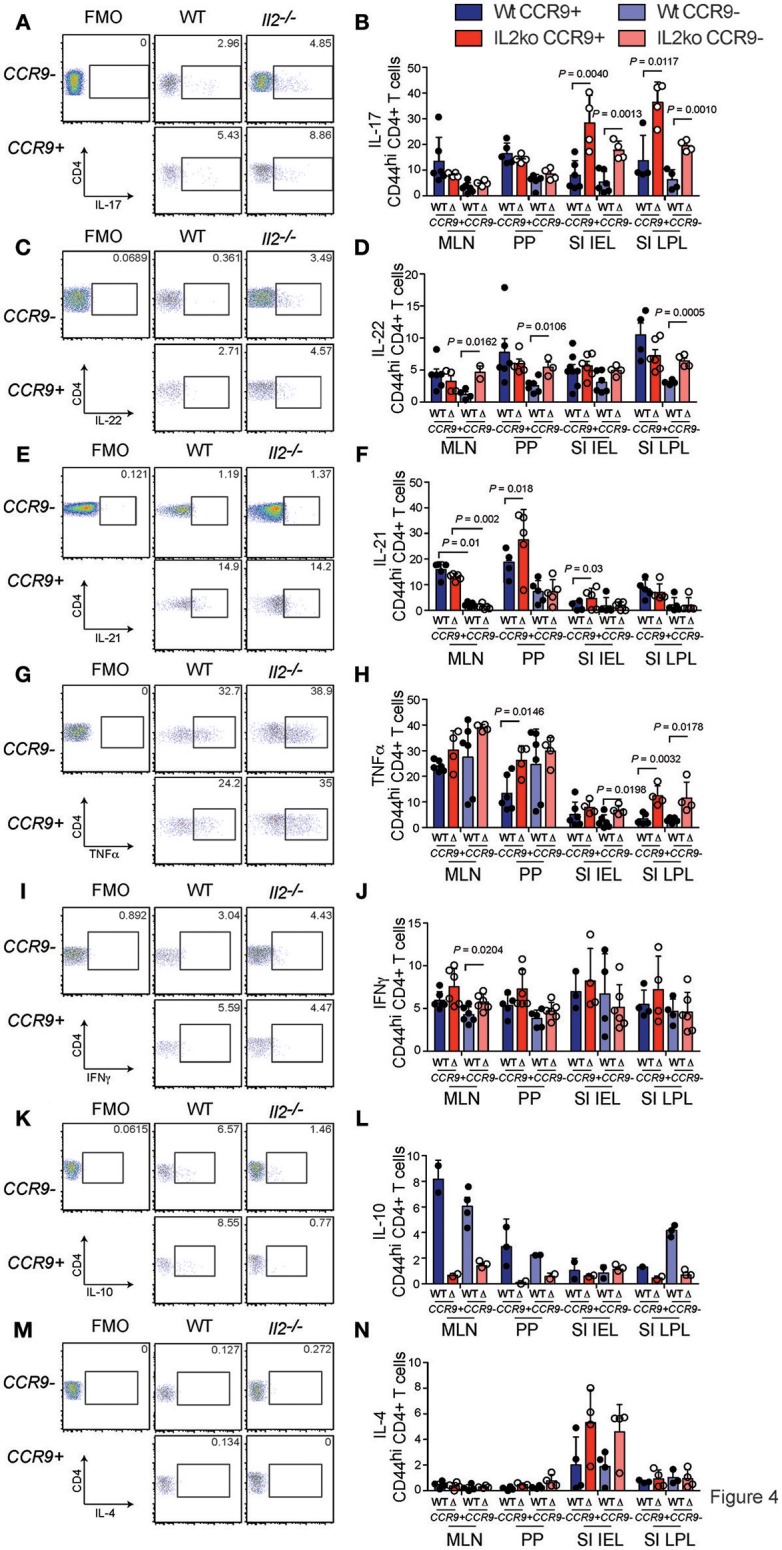
CCR9^+^ CD4^+^ Th cells exhibit a distinct cytokine profile in the inflamed GIT. Detection of cytokines in CCR9^+^ and CCR9^−^ Th cells from GIT associated lymphoid tissues of *Il2*^−/−^ and WT mice by intracellular immunostaining and FACS analyses. **(A)** Representative FACS dot plot and **(B)** quantitation of IL-17. **(C)** Representative FACS dot plot and **(D)** quantitation of IL-22. **(E)** Representative FACS dot plot and **(F)** quantitation of IL-21. **(G)** Representative FACS dot plot and **(H)** quantitation of TNFα. **(I)** Representative FACS dot plot and **(J)** quantitation of IFNγ. **(K)** Representative FACS dot plot and quantitation **(L)** of IL-10. **(M)** Representative FACS dot plot and **(N)** quantitation of IL-4. MLN, mesenteric lymph nodes; PP, Peyer's Patches; SI IEL, intraepithelial lymphocytes of the small intestine; SI LPL, lamina propria lymphocytes of the small intestine; LI IEL, intraepithelial lymphocytes of the large intestine; LI LPL, lamina propria lymphocytes of the large intestine. Data are shown as mean ± SD, where *n* = 3–6 female mice of 7–9 weeks of age. Statistical significance was analyzed by students *T*-test.

To further analyze the function of CCR9 expressing cells in the chronically inflamed GIT of *Il2*^−/−^ mice relative to IL-2 sufficient WT mice that do not develop GIT inflammation, we determined the expression of the pro-inflammatory cytokines (IL-17, IL-22, IL-21, TNFα, IFNγ) and the anti-inflammatory cytokines (IL-10, IL-4), which may be relevant to the chronic inflammation observed in *Il2*^−/−^ mice (29). One of the most striking features of CCR9^+^ Th cells in the GIT was the increased fraction of IL-17 producing cells. We observed a greater percentage of CD4^+^ T cells expressing IL-17 in *Il2*^−/−^ compared with WT mice, and a greater percentage of IL-17 producing Th cells were CCR9^+^ (Figures 4A,B). These findings indicate that IL-17 is commonly coexpressed with CCR9 on CD44^hi^ CD4^+^ T cells in the GIT. IL-22 also produced by Th17 cells, with both pro-inflammatory and regenerative functions. CD4^+^ T cells from *Il2*^−/−^ mice harbored a greater fraction of IL-22 producing cells than WT mice, but this was only significantly increased within the population of Th cells that lacked CCR9 expression (Figures 4C,D). The cytokine IL-21 is produced by both Tfh cells (33) and Th17 (34) cells, and CCR9^+^ IL-21-producing cells were also increased in both the MLN and PP of *Il2*^−/−^ mice relative to WT mice (Figures 4E,F), providing a source of IL-21 that may explain our previous observation of increased amounts of IL-21 in *Il2*^−/−^ mice (29).

Tumor necrosis factor (TNFα) is thought to contribute to the pathology of IBD in humans and mice (35). The percentages of TNFα-producing CCR9^+^ Th cells were significantly increased in the PP of *Il2*^−/−^ mice compared with WT mice (Figures 4G,H). An increased percentage of TNFα producing SI IEL was also observed in *Il2*^−/−^ mice, and the percentages of TNFα-producing SI LPL expressing CCR9 or lacking CCR9 were greater in the *Il2*^−/−^ mice (Figure 4D). Taken together, these findings indicate that there was not an associated coexpression with CCR9, but TNFα- expressing CD4^+^ T cells were related to the deficiency of IL-2 and to the chronic inflammation in these mice. By contrast, the percentages of IFNγ producing Th cells in *Il2*^−/−^ and WT mice were largely similar (Figures 4I,J). However, there was a significantly increased fraction of IFNγ ^+^ CCR9^−^ CD4^+^ cells in the MLN in *Il2*^−/−^ mice relative to the MLN of WT mice (Figures 4I,J). We also investigated the percentages of CD4^+^ T cells expressing the anti-inflammatory interleukins, IL-4 and IL-10. There was a marked trend of increased percentages of IL-10 expressing cells in the PP and MLN of WT mice compared to the *Il2*^−/−^ mice (Figures 4K,L). For IL-4, the fraction of CD4^+^ T cells expressing IL-4 was not significantly different between groups (Figures 4M,N).

**Figure 5:**
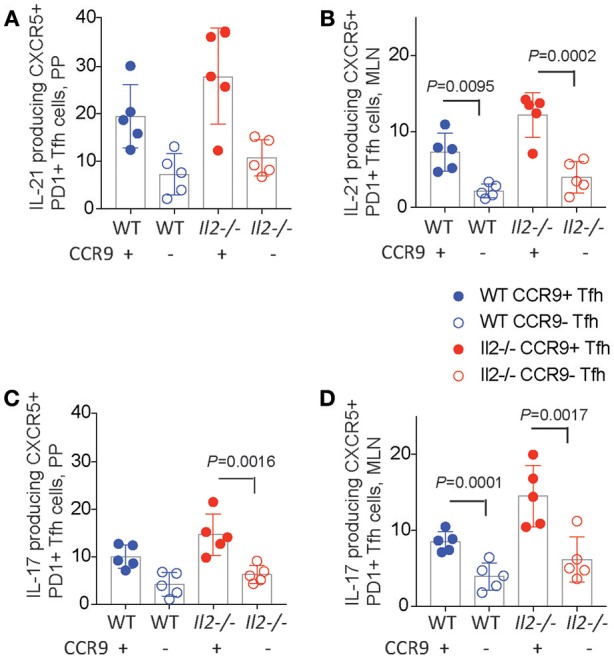
CCR9^+^ Tfh cells express increased amounts of IL-21 and IL-17. Percentages of cytokine expressing CCR9^+^ and CCR9^−^, CXCR5^+^ PD1^+^ CD4^+^ T follicular helper (Tfh) cells in the Peyers patches (PP) and mesenteric lymph nodes (MLN) of *Il2*^−/−^ and WT mice. Interleukin (IL) 17 and 21 were detected *ex vivo* by intracellualr immunostaining and FACs analyses. IL-21 containing CCR9^+^ and CCR9^−^ Th cells in the **(A)** PP and **(B)** MLN. IL-17 containing CCR9^+^ and CCR9^−^ Th cells in the **(C)** PP and **(D)** MLN. Data are shown as mean ± SD from 3 experiments, where *n* = 5 female mice of 9–12 weeks of age. Statistical significance was assessed by 2-way ANOVA using Bonferroni's multiple comparisons test.

Tfh cells have been reported to acquire effector functions associated with other T helper subsets, producing IL-17 (36, 37), IL-4 (38), and IFNγ in some studies, but not IL-17 (33, 39) or IL-4 (38) in others. We analyzed both CCR9^+^ and CCR9^−^ Tfh cells in the PP and MLN for the production of the cytokines IL-17 and IL-21 by intracellular immunostaining and FACS analyses. *Il2*^−/−^ mice contained the greatest percentages of IL-21 producing cells within the CCR9^+^ population in both the PP and MLN relative to CCR9^−^ Tfh cells (Figure 5B). Whereas, CCR9^+^ Tfh cells from WT mice contained more IL-21-producing cells in the MLN, but not the PP (Figures 5A,B). Intracellular detection of IL-17 demonstrated that CCR9^+^ Tfh cells from the PP of *Il2*^−/−^, but not WT, mice contained a greater percentage of IL-17 producing cells (Figure 5C). By contrast, the CCR9^+^ Tfh population also contained a greater percentage of IL-17 producing cells than the CCR9^−^ Tfh population in the MLN of both WT and *Il2*^−/−^ mice (Figure 5D).

### CCR9+ Th and Tfh Cells Exhibit a Site-Specific Transcriptome

Analyses of *Il2*^−/−^ mice indicated that CCR9^+^ Th cells and CCR9^+^ Tfh cells are phenotypically distinct from their CCR9^−^ counterparts. As discussed earlier, type-1 diabetes prone NOD mice harbor an increased number of IL-21-producing CCR9^+^ Th cells in the inflamed lesions of the pancreas and salivary glands that are phenotypically similar to Tfh cells (27). As CCR9 is a GIT-homing chemokine receptor, we questioned whether CCR9^+^ Th cells in the pancreas derive from CCR9^+^ Tfh cells in the GIT. Therefore, we determined the phenotypic relationship between CCR9^+^ Th and Tfh cells in the GIT and pancreas of NOD mice by differential gene expression analyses. RNA was extracted from FACs sorted CCR9^+^ and CCR9^−^, CXCR5^+^, PD-1^+^ TCRb^+^, CD4^+^ Tfh cells from the PP and from CCR9^+^ and CCR9^−^, CXCR5^−^, TCRb^+^, CD4^+^ cells from the pancreas. Gene expression was determined by SurePrint G3 Mouse GE 8x60K Microarray Kit from Agilent technologies and revealed a greater upregulation of genes in the CCR9^+^ populations. Therefore, we focused on the genes with increased expression in CCR9^+^ Th and Tfh cells in both tissues.

**Figure 6:**
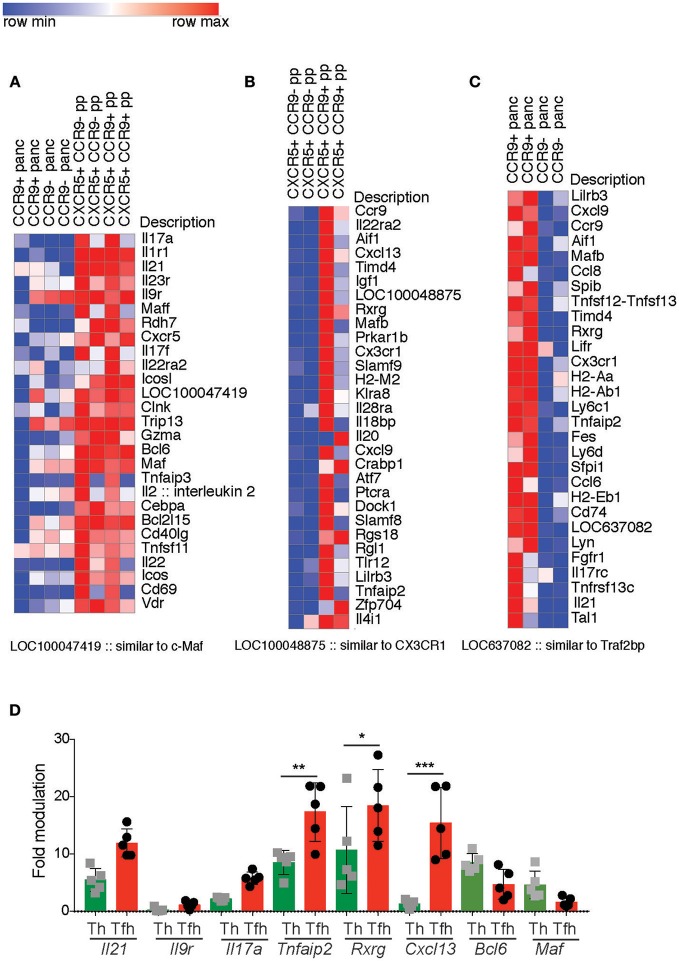
Differentially expressed genes in CCR9^+^ relative to CCR9^−^ Tfh from the peyers patches and CCR9^+^ relative to CCR9^−^ Th cells from the pancreas infiltrate from non-obese diabetic (NOD) mice. Gene expression was determined by SurePrint G3 Mouse GE 8x60K Microarray Kit from Agilent technologies. Genes selected from the 50 most differentially expressed (DE) genes shown in heat maps, Log2 Fold difference of 2.5–5.3 (fold difference of 5.6–34.6). **(A)** relatively higher expression of Th17 signature genes in CCR9^+^ T follicular helper (Tfh) cells from the Peyers patches compared with CCR9^+^ T helper (Th) cells from the pancreas of 10–12 week old female NOD mice. **(B)** DE genes from Peyers patch CCR9^+^ Tfh cells relative to Peyers patch CCR9^−^ Tfh cells. **(C)** DE genes from CCR9^+^ Th cells relative to CCR9^−^ Th cells from the pancreas infiltrate of 12 week old female NOD mice. **(D)** qPCR validation of DE genes selected from **(A–C)**. Gene expression of *Il21, Il9r, Il17a, Tnfaip2, Rxrg*, and *Cxcl13* from CCR9^+^ Tfh or Th cells analyzed by real-time PCR relative to Rpl19 expression. Data are shown as fold modulation of gene expression in CCR9^+^ Tfh relative to CCR9^−^ Tfh cells or CCR9^+^ Th cells relative to CCR9^−^ Th cells, where *n* = 5 mice per group. Statistical significance was assessed by 2-way ANOVA using Bonferroni's multiple comparisons test. *P < 0.05; **P < 0.01; ***P < 0.001.

The PP Tfh cell populations exhibited a clear Th17 transcriptome relative to the pancreas Th populations, indicating a microenvironment-specific signature (Figure 6A). Genes upregulated in the PP compared with the pancreas included genes known to be expressed in Tfh cells (as would be expected by a comparison of Th and Tfh cells) *Cxcr5, Icos, Bcl6, Maf, Il21* and Th17 signature genes *Il17a, Il21, Il23r, Il17f, Il22* (Figure 6A). Th17 signature genes were more enriched in CCR9^+^ Tfh cells relative to CCR9^−^ Tfh cells within the PP (Figure 6B). These data indicated that both CCR9^+^ and CCR9^−^ Tfh cells in the PP share characteristics of Th17 and Tfh genes, but also demonstrate notable differences; CCR9^+^ Tfh cells in the PP express increased amounts of *Ccr9, Il21, IL-22ra, v-Maf*, *Lifr, Cxcl13*, the cytokine and cytokine receptors *Il20, Il18bp, Il28ra* compared with CCR9^−^ Tfh cells in the PP (Figure 6B).

When we compared CCR9^+^ and CCR9^−^ Th cells in the pancreas, several of the most DE genes in CCR9^+^ cells at this site were amongst the most DE genes in CCR9^+^ Tfh cells in the PP. They included; *Ccr9, Cxcl9, Aif1, Rxrg, Cx3cr1, Lirb3, and Tnfaip2* (Figure 6C). Pancreatic CCR9^+^ Th cells were also distinct from their CCR9- counterparts in the pancreas by increased expression of genes known to be expressed by Tfh or Th17 cell, including *Il21, Il17rc, Fgfr1, Ccl6* (Figure 6C).

It was of interest to observe some clear similarities between the most differentially expressed genes in CCR9^+^ Th cells from the pancreas and in CCR9^+^ Tfh cells from the PP that suggested a GIT microenvironment, such as the retinoid receptor *Rxrg*, and Th17 cells or IL-17 interactions (Figures 6B,C). Taken together, these findings indicate that CCR9^+^ Th cells in the inflamed lesions of the pancreas in NOD mice are phenotypically related to GIT residing CCR9^+^ Tfh cells, supporting the notion that IL-21-producing CCR9^+^ Th cells that are critical for the development of T1D may emerge from Tfh cells in the GIT or a common GIT-residing precursor population.

**Figure 7:**
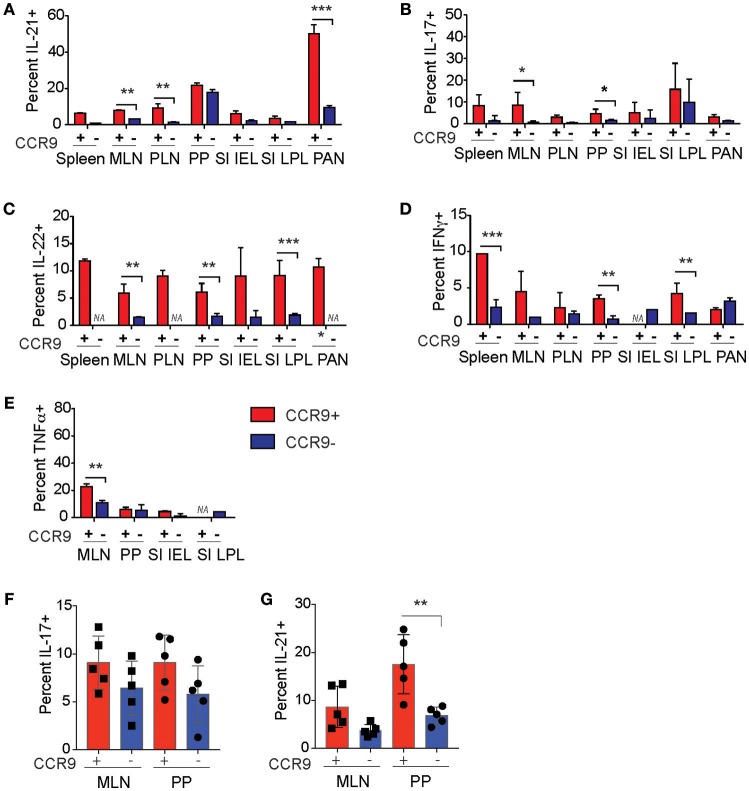
CCR9 marks populations of Th and Tfh cells with enhanced cytokine expression in autoimmune inflammation. Percentages of cytokine expressing CCR9^+^ and CCR9^−^ CD4^+^ T helper (Th) and T follicular helper (Tfh) cells in the GIT and inflamed pancreas of NOD mice. CCR9^+^ and CCR9^−^ Th cells that contain **(A)** IL-21, **(B)** IL-17, **(C)** IL-22, **(D)** IFNγ, and **(E)** TNFα in the spleen. MLN, mesenteric lymph nodes; PP Peyer's Patches; SI IEL, intraepithelial lymphocytes of the small intestine; SI LPL, lamina propria lymphocytes of the small intestine; LI IEL, intraepithelial lymphocytes of the large intestine; LI LPL, lamina propria lymphocytes of NOD mice. Percentages of **(F)** IL-17 and **(G)** IL-21 expressing CCR9^+^ and CCR9^−^ Tfh cells in the mesenteric lymph nodes (MLN) and Peyers patches (PP) of NOD mice. Cytokines were detected by intracellualr immunostaining and FACs analyses. Data is shown as mean ± SD from 3 experiments, female mice 11–12 weeks of age *n* = 5/group for Tfh cell analyses and *n* = 7/group for Th cell analyses. Statistical significance was assessed by 2-way ANOVA using Bonferroni's multiple comparisons test. *P < 0.05; **P < 0.01; ***P < 0.001.

The results from our microarray indicated some variation between samples that may reflect differences in inflammation and the disease progression between individual female NOD mice. Therefore, to validate our findings we performed qPCR on selected genes in CCR9^+^ and CCR9^−^ populations from the PP and pancreas (Figure 7). *Il21, Cxcl13, Rxrg, Il17a*. The expression of genes by qPCR was normalized based on the level of the housekeeping gene *Rpl19* and shown as fold modulation of CCR9^+^ Th and Tfh cells relative to CCR9^−^ Th and Tfh cells, respectively. *Il21* was increased in both CCR9^+^ Th and Tfh cells compared with CCR9^−^ cells, where it was most highly expressed in the CCR9^+^ Tfh population (Figure 6D). *Tnfaip2, Rxrg* and *Cxcl13* were also significantly increased in both CCR9^+^ Th and CCR9^+^ Tfh cells compared with their CCR9^−^ counterparts (Figure 6D). By contrast, expression of *Il9r* was not increased in either CCR9^+^ populations compared with CCR9^−^ cells (Figure 6D). Taken together, these data support our microarray findings and show that CCR9^+^ distinguishes both Th and Tfh cell populations.

### CCR9^+^ Th and Tfh Cells Exhibit a Site-Specific Phenotype

Flow cytometric analyses of CCR9^+^ and CCR9^−^ Th and Tfh cell populations in the lymphoid tissues of the GIT, pancreas and pancreatic lymph nodes in NOD mice revealed that an increased percentage of CCR9^+^ cells in NOD mice express an array of cytokines in the GIT relative to CCR9^−^ cells. CCR9^+^ Th cells from NOD mice contained a greater fraction of IL-21 producing cells in the spleen, MLN, PLN and pancreas compared with CCR9- Th cells (Figure 7A). IL-17 producing cells were concentrated in the CCR9^+^ populations in the spleen, MLN and PP (Figure 7B). CD4^+^ Th cells from NOD mice exhibited a greater fraction of IL-22 producing cells than WT mice in all tissues, significantly increased within the MLN, PP and LPL populations (Figure 7C). CCR9^+^ Th cells in the MLN, PP, and SI LPL contained the greatest percentages of IL-22 producing cells (Figure 7C). In addition, there was an increased fraction of IFNγ-producing CCR9^+^ Th cells in the spleen, PP and SI LPL (Figure 7D). By contrast, CCR9^−^ Th cells contained a greater fraction of IFNγ producing cells in the pancreas compared with CCR9^+^ Th cells (Figure 7D). TNFα-producing cells, in turn, were enriched in the CCR9^+^ Th fraction in the MLN alone of NOD mice (Figure 7E).

Similar to the cytokine profile of CCR9^+^ Th cells; CCR9 expression distinguished the Tfh population with the highest fraction of both IL-17 (Figure 7F) and IL-21 (Figure 7G). Taken together, these findings are consistent with our microarray findings and suggest that CCR9-expressing Th and Tfh cells contain differentiated/effector cells that express an array of cytokines, notably cytokines that are associated with Th17-like cells and differentiated Th cells within the GIT microenvironment.

### CCR9+ Tfh and CCR9+ Th Cells From GIT Lymphoid Tissues Migrate to the Pancreas

**Figure 8:**
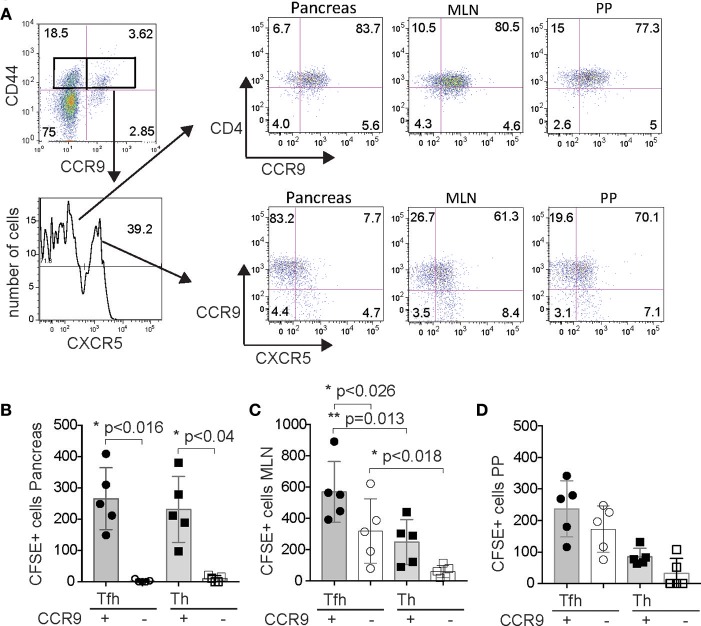
CCR9 marks GIT-derived Tfh cells and Th cells that migrate to the pancreas. **(A)** FACS sorting strategy showing representative FACS dot plots gated on CD4 and then CD44 vs. CCR9 and FACS histogram of CXCR5 expression for purification of CCR9^+^ CXCR5^+^ Tfh cells, CCR9^−^ CXCR5^+^ Tfh cells, CCR9^+^ CXCR5^−^ Th cells and CCR9^−^ CXCR5^−^ Th cells from the PP and MLN, and representative FACS dot plots of CCR9^+/−^ Th cells and CCR9^+/−^ Tfh cells from the pancreas infiltrate, mesenteric lymph nodes (MLN) and Peyers patches (PP). 6 × 10^5^ CFSE labeled cells from each of the 4 subsets were injected (i.v.) into age matched female NOD recipients (*n* = 5/transfer group). Quantitation showing numbers of CFSE labeled CD4^+^ CCR9^+^ and CD4^+^ CCR9^−^ Tfh and Th cells recovered from the **(B)** pancreas, **(C)** mesenteric lymph nodes and **(D)** Peyers patches of NOD mice 4 days after adoptive transfer. Statistical significance was assessed by 2-way ANOVA using Bonferroni's multiple comparisons test.

Previous analyses of CCR9^+^ Th cells from the inflamed pancreas of pre-diabetic NOD mice indicated some phenotypic similarities with Tfh cells and we hypothesized that CCR9^+^ Th cells may emerge from Tfh-like cells in GIT lymphoid tissue (27). To test whether Tfh cells from GIT-associated lymphoid tissue could migrate to the pancreas, we performed an adoptive transfer experiment using FACS sorted CFSE labeled cell subsets. CCR9^+^ Tfh cells, CCR9^−^ Tfh cells, CCR9^+^ Th cells, and CCR9^−^ Th cells were sorted from the PP and MLN of 12-14 week old female NOD mice (Figure 8A), CFSE labeled and then transferred into 12 week old (pre-diabetic) NOD female recipients. Four days later, CFSE^+^ cells were recovered from the PP, MLN and pancreas and analyzed by immunostaining for CXCR5 and CCR9 by FACS. The results show that CCR9^+^ Th cells from the GIT lymphoid tissues migrated to and accumulated in small numbers in the pancreas (Figure 8B) the MLN (Figure 8C) and the PP (Figure 8D). By contrast, Th cells that lacked expression of CCR9 migrated to or accumulted poorly at all three sites.

The expression of CCR9 was similarly important for Tfh cells from the PP and MLN that had migrated into the pancreas, which were predominantly those that had lost expression of CXCR5, but had retained CCR9 (Figure 8B). It is unlikely that the Tfh cells had lost CFSE due to proliferation as we have observed few endogenous Tfh cells in the pancreas (27). Previous studies have shown that Tfh cells maintain plasticity and can downregulate CXCR5 (40) and those data are consistent with our findings. By contrast, adoptively transferred CFSE^+^ Tfh cells that were retrieved from the PP and MLN contained a mixture of phenotypes. Four days after the CCR9^+^ Tfh cells transfers, we observed cells that expressed both CXCR5 and CCR9, as well as cells expressing reduced levels of both molecules (Figure 8A). CXCR5^+^ CCR9^+^ Tfh cells were retrieved in greater numbers from the MLN than CCR9^−^ (CXCR5^+^) Tfh cells or CCR9^+^ Th cells (Figure 8C). By contrast, whilst there was a trend of increased recovery of Tfh cells from the PP, there were no significant differences observed between the recovery of each of the 4 populations from the PP (Figure 8D). These findings demonstrate that Tfh cells from the GIT associated lymphoid tissues retain plasticity and that the expression of CCR9 is important for the migration of GIT lymphoid tissue Tfh cells and Th cells from the bloodstream into the pancreas.

## Discussion

Previous examination of the phenotype of CCR9+ Th cells in the inflamed lesions of the pancreas of T1D-prone NOD mice suggested a close relationship between CCR9^+^ Th cells and Tfh cells and we hypothesized that CCR9^+^ Th cells may emerge from Tfh-like cells in GIT lymphoid tissue (27).

Using a combination of gene expression analyses and flow cytometric analyses of cells in the GIT and GIT associated lymphoid tissues, this study demonstrates a microenvironment specific signature for Tfh cells and Th cells that express the gut-homing chemokine receptor CCR9. We demonstrate that the expression of CCR9 marks the Tfh cells (and Th cells) that derive from the peyers patches and mesenteric lymph nodes that can migrate into the inflamed lesions of the pancreas in NOD mice.

Several studies have demonstrated that lymphocytes infiltrating the islets in T1D humans and the NOD mouse express α4β7-integrin, supporting a link between T1D and the gastrointestinal immune system (41–44). In addition, antibodies blocking α4β7 or MadCAM-1, prevent diabetes in NOD mice (15). The findings presented here reveal a closer association between the phenotype and transcriptome of Tfh cells that express the GIT homing receptor CCR9 and CCR9^+^ Th cells in the inflamed pancreas of NOD mice (27) than was previously appreciated. CCR9^+^ Tfh cells in the GIT and CCR9^+^ Th cells in the pancreas express common gene sets and express greater amounts of cytokines, such as IL-21 and IL-17, than their CCR9^−^ counterparts at the same sites. The production of IL-21 is a feature of both CCR9^+^ Th and CCR9^+^ Tfh cells, which is critical for Th cells to provide help to both CD8^+^ T cells and B cells.

High levels of CCR9 have previously been detected in SI lymphocytes (45) and in the small bowel during IBD (46). Here, we analyzed the characteristics of Tfh cells and Th cells that express CCR9 in the GIT associated lymphoid tissues of *Il2*^−/−^ mice that exhibit chronic inflammation in the GIT. The expression of CCR9 marked a population of Tfh cells and Th cells with an increased capacity for cytokine production, which may suggest an increased level of activation or differentiation. CCR9^+^ Th cells in the inflamed GIT of *Il2*^−/−^ mice additionally exhibited increased expression of CCR6 and α4β7 compared with their CCR9^−^ Th counterparts. Co-expression of the integrin α4β7 and CCR9 further emphasizes the GIT-specific nature of CCR9^+^ Th cells as α4β7 specifies the recruitment of T cells to the intestinal mucosa through its interaction with its ligand MAdCAM-1, where it is critical in a TNFα-dependent model of CD (47). CCR6, in turn, is expressed on Th17 cells and has been associated with CD (48).

Co-expression analyses of CCR9 and cytokine production demonstrated an increased expression of cytokines that are typically produced by Th17 cells in both CCR9^+^ Th and Tfh cells in *Il2*^−/−^ mice compared to these populations in WT mice. Our findings indicate that IL-17 is commonly coexpressed with CCR9 on CD44^hi^ CD4^+^ T cells in the GIT, which contrasts with the lack of IL-17 production in CCR9^+^ Th cells purified from the inflamed pancreas of NOD mice (27). CCR9^+^ T cells express pro-inflammatory cytokines in both CD and UC (26, 49, 50). Whilst our findings are consistent with increased production of IL-17 from CCR9^+^ T cells isolated from CD LPL (50) we did not observe increased IFNγ as shown previously in CD in humans (50). TNFα was also produced by a greater fraction of PP and SI LPL CCR9^+^ Th cells in the inflamed GIT of *Il2*^−/−^ mice compared with WT mice, which is consistent with the inflammatory environment. Taken together, our findings demonstrate an increase in the production of pro-inflammatory cytokines, especially by CCR9^+^ Th and Tfh cells, in the inflamed GIT.

The expression of the GIT-homing chemokine receptor CCR9 distinguished populations in both the inflamed lesions of the pancreas and GIT associated lymphoid tissues. We have previously shown that NOD mice contain small numbers of IFNγ- producing CD4^+^ T cells in the pancreatic islet lesion, but they were largely restricted to the CCR9^−^ population (27). By contrast, CCR9^+^ Th cells from the GIT associated lymphoid tissues of NOD mice produced more cytokines than CCR9^−^ Th cells, including the Th1 cytokines TNFα and IFNγ, and the Th17 cytokines IL-17, IL-21, and IL-22. The microenvironment of the GIT favored a Th17-like phenotype in Tfh cells from both *Il2*^−/−^ and T1D-prone NOD mice. Indeed, the transcriptome of Tfh cells from the PP of NOD mice contained both Tfh and Th17 signature genes that were not expressed in Th cells from the inflamed pancreas. High levels of IL-17 have been previously reported in the colon of young NOD mice, which may reflect GIT inflammation (28). When CCR9^+^ cells were compared with their CCR9^−^ counterparts at each site; CCR9^+^ Tfh cells from the PP and CCR9^+^ Th cells from the pancreas shared genes that were among the most DE at both sites, including *Ccr9*, genes induced by cytokines, interferons and retinoic acid such as *Cxcl9, Cx3cr1, Aif1, and Tnfaip2* (51), *Lilrb3*, which binds MHC1 to transduce a negative signal (52) and a component of the retinoid receptor *Rxrg* (53), which is consistent with the GIT microenviroment where retinoic actid is produced by CD103 expressing dendritic cells (54).

The ligand for CCR9 is Chemokine (C-C motif) ligand 25 (CCL25), which is expressed in the pancreas of pre-diabetic NOD mice (27). Similarly, inflammation can increase the expression of CCL25 in colitis where CCL25 expression correlates with inflammation in the GIT (55). In this manner, the inflamed lesions of the pancreas observed in NOD mice may serve to attract CCR9^+^ Tfh cells from the GIT and GIT-associated lymphoid tissues, or CCR9^+^ Th cells from the GIT that acquire Tfh characteristics in the pancreas or pancreatic lymph nodes, to participate in the destruction of self-tissue in the pancreas and development of autoimmunity.

Data derived from several clinical studies suggest that pharmaceutical CCR9 small-molecule inhibitors have beneficial effects in patients with CD (56, 57). The observation of organ-specific CCR9-mediated homing of activated T cells to the intestine renders CCR9 a prime therapeutic target to inhibit the recruitment of immune cells whilst avoiding global immune suppression. A greater understanding of the CCR9 expressing Th cells in immunity and autoimmunity will facilitate the generation of new strategies for the treatment of IBD and inflammatory diseases that affect the accessory organs of the digestive system.

## Materials and Methods

### Mice

Female NOD Ltj mice were obtained from ARC, Perth, WA. C57BL/6 *Il2*^−/−^ mice were purchased from Jackson Laboratories (ME, USA) and backcrossed to C57BL/6 to N12.

### Immunohistochemistry

Five micrometer sections of paraffin-embedded intestine were conventionally stained with haematoxylin and eosin (H&E) for histological evaluation. Sections were Analyzed using a Leica light microscope (Leica Microsystems, Wetzlar, Germany). The images were processed using the Leica acquisition and analysis software ImageJ (Freeware NIH Bethesda, USA) and Adobe Photoshop, version 7 (San José, CA).

### Flow Cytometry; Surface Immunostaining

Spleen and lymph nodes were homogenized using 70 μm cells strainers in lymphocyte isolation buffer. For flow cytometric analysis of pancreas infiltrate, intraepithelial lymphocytes (IELs) and lamina propria lymphocytes (LPLs), small intestine samples were subjected to lymphocyte isolation as described in detail below. Red blood cells (RBC) were removed from spleens using 2 ml RBC lysis buffer for 1 min on ice before washing in lymphocyte isolation buffer. Fifty microliter of a single cell suspension at 2 × 10^7^ cells/ml from spleen and lymph nodes were stained in FACS buffer containing pre-titred antibodies in 96 well V-bottomed microtitre plates (Nunc, Roskilde, Denmark) at concentrations shown in Table 1. To reduce non-specific binding, cells were pre-treated with anti-CD16 for 20 min (2.4G2 made in house). Cells were acquired using Canto cytometer (BD Biosciences, CA) and analyzed using Flowjo (Treestar, CA). Doublets were excluded by forward scatter height and width. Data was collected on a Canto flow cytometer (BD Biosciences), and analyzed using FlowJo software (Tree Star, Inc.).

**Table 1:** Antibodies for Flow Cytometry.

**Molecule**	**Clone (company)**	**Label**	**Dilution**
CCR6	140706 (BD Biosciences)	Alexa Fluor 647	1:200
CCR9	eBioCW-1.2 (eBioscience)	FITC	1:150
CD122	PO3.1 (eBioscience)	PE	1:100
CD127	A7R34 (eBioscience)	APC	1:200
CD3	145-2C11 (BD Biosciences)	FITC	1:200
		Pacific Blue	1:200
CD4	RM4-5 (BD Biosciences)	APC	1:400
		Alexa Fluor 750	1:300
		Pacific Blue	1:300
CD44	IM7 (eBioscience)	FITC	1:200
		APC	1:200
CD45.2	A20 (eBioscience)	PeCy7	1:300
CD69	H1.2F3 (BD Biosciences)	FITC	1:200
CXCR5	2G8 (BD Biosciences)	Biotin	1:100
IFNγ	XMG1.2 (eBioscience)	FITC	1:200
		Pacific Blue	1:100
IL-10	JES5-16E3 (eBioscience)	APC	1:200
IL-17A	TC11-18H10 (eBioscience)	FITC	1:100
IL-21	Polyclonal (R&D Systems)	Biotin	1:100
IL-22	142928 (R&D Systems)	PE	1:100
IL-4	BVD6-24G2 (eBioscience)	FITC	1:100
IL-9R	224325 (R&D Systems)	PE	1:200
PD-1	J43 (BD Biosciences)	FITC	1:100
		PE	1:100
TNFα	MP6-XT22 (BD Biosciences)	PE	1:200
α4β7	DATK32 (BD Biosciences)	PE	1:200

### Flow Cytometry; Intracellular Immunostaining for Detection of Cytokines

Intracellular cytokines were detected using the BD sciences intracellular staining kit according to the manufacturer's instructions. Cytokines were detected either directly *ex-vivo* or after 4 h stimulation at 37°C in cell culture media with PMA (50 ng/ml, BIOMOL), ionomycin (500 ng/ml, Invitrogen) and GolgiPlug (1:1000, BD Biosciences) following *ex vivo* staining of surface markers, cells were fixed and permeated (BD Biosciences), followed by intracellular staining with antibodies at concentrations shown in Table 1 (58).

### FACS Sorting of CCR9^+^ and CCR9^−^ Tfh and Th Cells

For sorting of CCR9^+/−^ Tfh and Th cells for microarray analyses (FACSAria cell sorter), we gated on lymphocytes, CD4^+^ T cells by CD4 expression, and then subdivided by CD44 and CCR9. In this way we circumvented staining CD3 by gating lymphocytes to avoid potentially activating the cells being sorted. RNA was extracted from cells with an RNeasy Mini Kit (QIAGEN).

### Real-Time qPCR

RNA was obtained from cells with an RNeasy Mini Kit (QIAGEN) and cDNA synthesized with the SuperScript III First-Strand Synthesis System (Life Technologies). We determined the relative abundance of cDNAs in triplicate by qRT-PCR analysis using the Light Cycler 480 (Roche). Fluorescence signals were measured over 45 PCR cycles (94°C for 15 s, 60°C for 30 s, 72°C for 15 s) and the cycle (Ct) at which signals crossed a threshold set within the logarithmic phase was recorded. For each assay, standard curves were generated to identify positive signals on the linear part of the curve.Real-time pre-designed PCR primer pairs for mouse genes were obtained from Applied Biosystems and probes from Roche. Housekeeping gene: Rpl19 F ccacaagctcttttcctttcg Roche probe #46, Rpl19 R ggatccaaccagaccttcttt Roche probe #46. For quantitative real-time PCR (qRT-PCR), 200 ng RNA was treated with DNase I (Qiagen) for 30 min at 37°C, before cDNA synthesis. Relative gene expression values were normalized based on the level of the housekeeping gene Rpl19 and calculated using the relative gene quantification tool from the LightCycler 480 software (v.1.5, Roche). Fold modulation of mRNA was calculated by employing a comparative Ct method; Relative abundance of genes = 2(ΔCt), where ΔCt is the difference between the Ct of target and the arithmetic mean of Cts of Rpl19.

### Pancreas Infiltrate Isolation

Mice were perfused with PBS, and pancreas extracted (27). Pancreas were cut into small pieces with scissors and transferred into 50 ml falcon tubes with 3 mls of 0.25 mg/ml Liberase-Enzyme Blend-RI (Roche) in serum free RPMI 1640 media. The tissue was digested in a 37°C water bath for 20 min. Tubes were centrifuged at 201 g at 4°C for 5 min and the supernatant discarded. ten ml of cold serum containing (10%) RPMI 1640 media was added. Tubes were vortexed and shaken to dislodge the tissue; centrifuged at 201 g at 4°C for 5 min and the supernatant discarded. Again the supernatant was discarded, and the tissue resuspended in 5 ml serum-free RPMI 1640, centrifuged at 201 g at 4°C for 5 min. Pellets were thoroughly resuspended in 10 ml histopaque (Sigma-Aldrich) by vortexing. 5 ml of serum-free RPMI 1640 was layered on top. The tubes were centrifuged at 974 *g* at 4°C for 10 min without rotor acceleration or deceleration Pancreatic infiltrating lymphocytes at the media:histopaque interface were collected, and transferred into a new 15 ml tube. Tubes were the centrifuged at 340 *g* at 4°C for 5 min, the supernatant discarded, the pellet washed in 5 ml PBS and centrifuged again. To dislodge clumped cells, samples were resuspended in 1 ml x for 1 min and washed in serum containing RPMI 1640.

### LPL and IEL Isolation

Small intestines were extracted from mice and placed in a petri dish containing pre-warmed PBS. Peyer's patches were removed and the small intestine was cut longitudinally to allow feces to be washed away. Samples were cut into small pieces with scissors and transferred into 50 ml falcon tubes and washed until media cleared.

To isolate intraepithelial lymphocytes (IEL), the tissues were incubated in 20 ml of IEL stripping buffer for 20 min at 37°C while shaking. Tissues were allowed to settle and the supernatant decanted through a cell strainer, then washed twice in lymphocyte isolation media and suspended in 8 ml of 40% Percoll (GE Healthcare). Three microliters of 70% Percol was underlayed using a glass pipette and the sample was centrifuged at 600 g for 20 min at room temperature. The IEL were then removed from the resulting interface, washed twice in lymphocyte isolation media by centrifuging at 300 *g* for 5 min at 4°C and used immediately for flow cytometry analysis.

To isolate the lamina propria lymphocytes (LPL), the tissue remaining after treatment with stripping buffer was washed twice as above and resuspended in 5 ml of 5 mg/ml collagenase D (Roche) and 0.05% DNAse (Promega) in lymphocyte isolation media. Tissues were incubated in the enzyme solution for 15 min at 37°C then another 10 mL were added and incubated for another 15 min. Tissues were removed and washed twice as above, then passed through a 70 μm cell strainer. These cells were run on a Percoll gradient as above then used immediately for analysis.

### Adoptive Transfer of CCR9^+/−^ Tfh and Th Cells

CCR9^+/−^ Tfh and Th cells were sorted (FACSAria cell sorter) for adoptive transfer from combined MLN and peyers patches by gating on lymphocytes then CD4^+^ CD44^+^ cells, CCR9^+^ or CCR9^−^ cells and CXCR5 to denote Tfh cells. For CFSE labeling; cells were washed twice in PBS and resuspended at 5 × 10^7^ per ml for CFSE staining in CFSE buffer containing 5 mM CFSE. Cells were incubated at 37°C for 10 min then washed twice with ice cold lymphocyte isolation media before being prepared for adoptive transfer. Twelve week-old female NOD mice were administered 6 × 10^5^ (i.v.) of CFSE labeled CCR9^+^ Tfh cells, CCR9^−^ Tfh cells, CCR9^+^ Th cells or CCR9^−^ Th cells and recovery of CFSE^+^ cells from the MLN, PP and pancreas infiltrate was determined by immunostaining, flow cytometry and FACS analyses on day 4.

### Data Analyses and Statistics

*P*-values were determined by either students *T*-test or 2-way ANOVA using Bonferroni's multiple comparisons test. Data are reported as the mean ± standard deviation (SD), along with the calculated *P*-values.

## Ethics Statement

Animals were housed under specific pathogen-free conditions and handled in accordance with the Garvan Institute of Medical Research and St. Vincent's Hospital Animal Experimentation and Ethics Committee, which comply with the Australian code of practice for the care and use of animals for scientific purposes.

## Author Contributions

IC and HM performed the experiments, analyzed the data, and prepared the figures. JW performed the experiments. MD was involved in discussion about the work and planning of the experiments. CK directed the research, analyzed the data, created the figures, and wrote the manuscript.

### Conflict of Interest Statement

The authors declare that the research was conducted in the absence of any commercial or financial relationships that could be construed as a potential conflict of interest.
